# Association between Air Quality and Sedentary Time in 3270 Chinese Adults: Application of a Novel Technology for Posture Determination

**DOI:** 10.3390/jcm7090257

**Published:** 2018-09-06

**Authors:** Yiqun Ma, Bing Yuan, Shuhui Fan, Yizhou Luo, Xu Wen

**Affiliations:** 1 Department of Sport & Exercise Science, College of Education, Zhejiang University, Hangzhou 310028, China; mayiqun@zju.edu.cn (Y.M.); fsh1130@zju.edu.cn (S.F.); luoyizhou@zju.edu.cn (Y.L.); 2 Information Technology Center, Zhejiang University, Hangzhou 310028, China; yuanbing@zju.edu.cn

**Keywords:** sedentary behaviour, air quality, socio-ecological model, wrist-worn activity tracker

## Abstract

This study investigated the association between ambient air quality and sedentary time in Chinese adults. The participants were 3270 Chinese users (2021 men and 1249 women) of wrist-worn activity trackers. The data of participants’ daily activities were collected from July 2015 to October 2015. A novel algorithm based on raw accelerometer data was employed to determine sedentary time. Personal data, including sex, age, weight and height, were self-reported by the participants. Data of air quality, ambient temperature and weather were collected from the data released by the China National Environmental Monitoring Centre and the China Central Meteorological Observatory and matched in accordance with the Global Positioning System and time information. Multilevel regression analyses were conducted to investigate the association between air quality and sedentary time and adjusted for gender, age, region, body mass index, weather, temperature, weekday/weekend and monitored wake time per day. Better air quality index levels and lower concentrations of fine particulate matter were significantly associated with approximately 20 and 45 min reduction in sedentary time, respectively. Poor air quality appears to be an independent factor associated with prolonged sedentary time in Chinese adults.

## 1. Introduction

Sedentary behaviour is defined as any waking behaviour with a sitting, reclining or lying posture that requires an energy expenditure ≤1.5 metabolic equivalents [1]. It is a distinct concept from physical inactivity, which refers to an insufficient physical activity level to meet the present physical activity recommendations [1]. Sufficient moderate- to vigorous-intensity physical activity (MVPA) means that an individual is not physically inactive but cannot guarantee the individual from accumulating a large amount of sedentary time. Several studies have reported that the negative health consequences of prolonged sedentary time may include obesity, type 2 diabetes and cardiovascular diseases (e.g., coronary heart disease, myocardial infarction and stroke) [2,3,4]. These consequences are suggested to be independent of those attributable to the lack of physical activity [5,6,7]. Moreover, strong evidence suggests that people who engage in high amounts of sedentary behaviour can be at increased risk of mortality regardless of their level of MVPA [6,8,9]. Although a study based on 155,054 Chinese adults showed that 81.3% of the men and 88.5% of the women were physically inactive [10], few studies have reported the sedentary time of Chinese adults. Therefore, given the limited data on sedentary behaviour in Chinese adults and the usually negative health consequences of sedentariness, investigations of sedentary time in China should be made, and correlates should be clarified to help design, tailor and evaluate sedentary behaviour interventions.

A socio-ecological model is widely applied in the conceptualisation of factors influencing sedentary behaviour [11,12,13]. This socio-ecological approach assumes that multiple levels of influence, including intrapersonal, interpersonal, organisational, environmental and policy, exist, thus emphasising the interrelationships between individuals and the social, physical and policy environments [13]. A large number of factors in the model, particularly individual correlates, have been verified by numerous empirical studies [12,14]. In terms of physical environmental factors, type of residence [15,16], density [17] and proximity [18] of green spaces; neighbourhood walkability [19,20]; and season [21] have been reported to be related to sedentary behaviour. To date, however, the associations between other physical environmental factors such as air quality with sedentary behaviour have not yet been sufficiently investigated.

Although air quality has generally improved in China, the current situation of air pollution in the country is still a cause for concern. In 2016, the annual mean concentration of fine particulate matter (PM_2.5_) was 47 µg/m^3^ [22], which is 14.8% higher than the Chinese primary standard (40 µg/m^3^) [23] and 213.33% higher than the American secondary standard (15 µg/m^3^) [24]. Although the detrimental impact of air pollution on human health has been universally proven [25,26], knowledge about its effect on health behaviour is limited.

Several studies have investigated the association between air pollution and physical inactivity. These studies have shown that poor air quality may lead to decreased physical activity. Using data from the Behavioural Risk Factor Surveillance System (BRFSS), Wen et al. examined the relationship between air quality index (AQI) media alerts and changes in outdoor activities amongst people with asthma and showed that media alerts may be associated with self-reported decreases in outdoor activities [27]. Two other cross-sectional studies based on data from the BRFSS indicated that increased levels of air pollution are related to reduced leisure-time physical activity amongst American adults [28,29]. This result was confirmed by a retrospective study in the United States, in which ambient PM_2.5_ air pollution was observed to be associated with a modest but measurable increase in the leisure-time physical inactivity of individuals [30]. Similar findings amongst Chinese adults have been made. By analysing the data collected from 153 users of an exercise app and the AQI during the same period, Hu et al. concluded that people’s participation in outdoor exercise was impeded by air pollution severity [31]. Cohort studies of university retirees [32] and university students [33] living in Beijing, China, also revealed the same conclusions.

Despite the abundant literature concerning the relationship between air quality and decreased physical activity, we only found one empirical study involving the association between air quality and sedentary behaviour. In a population-based mail survey of 1332 Australian adults, researchers examined the associations between physical activity and sedentary behaviour with multiple individual-level and environmental-level variables; one of their findings was that air pollution was significantly associated with increased sedentary time [34].

Considerable attention has been paid on the measurement of sedentary behaviour. Subjective methods, including self-report or proxy-report questionnaire, are widely applied in studies of sedentary behaviour; however, the validity and reliability of these responses are subject to recall error and social desirability bias [35]. For objective methods, accelerometers can be used to estimate the total time and breaks of sedentary behaviour [36], which are based on movement detection but are traditionally considered to be poorly able to distinguish amongst different postures, therefore misestimating the real sedentary time [37]. Accelerometers are also typically hip-worn devices, which leads to lower wear compliance than wrist-worn devices [38]. Sedentary Sphere, a novel algorithm, was developed to analyse data from wrist-worn triaxial accelerometers and determine postures, leading to better compliance in large samples [39]. By using the method, the direction of the activity monitor and the wrist position could be determined based on the gravitational component of the acceleration data. In brief, posture is determined according to the angle of arm elevation and the intensity of activities. For instance, if arm elevation is higher than 15° below the horizontal position and the intensity of activity is light or moderate, the data indicate a seated or reclining position and could be identified as sedentary behaviours. This method was found to be valid in measuring sedentary time [40]. Moreover, as Sedentary Sphere is mainly based on the direction of the gravity component rather than the magnitude of accelerations, it could be applied to estimate sedentary time with different brands of wrist-worn accelerometers in different studies [41]. In addition, the cost of commercial activity monitors with Sedentary Sphere is lower than that of professional monitors, such as activPAL, therefore allowing a relatively larger sample size.

In the present study, a popular brand of activity tracker named Bong II (which is a light, convenient and wrist-worn triaxial accelerometer with Sedentary Sphere algorithm) was used to determine whether the sedentary time of Chinese adults is associated with ambient air quality. We hypothesised that people would spend more sedentary time in days with bad air quality than in days with good air quality.

## 2. Methods

### 2.1. Participants

In this study, researchers contacted several manufacturers of wrist-worn accelerometers, introduced the research plan and sought for research cooperation. Hangzhou Gongke Technology Co., Ltd., the manufacturer of the Bong II accelerometer, agreed to participate. With the assistance of the manufacturer, an informed consent form that introduced the objective and content of the research as well as the benefits of participating in the study was sent to approximately 20,000 customers via the Internet. The participants would receive a personal sedentary behaviour report for free. Only users who endorsed the informed consent form were recruited to this study. The data were collected from the Bong II activity monitors and transmitted to the company via the Internet. Personal information, including age, sex, height and weight, was self-reported on the App of Bong II by participants when they first used the product. Researchers were unable to access the data until privacy information (name, address, telephone number, email, etc.) of participants was deleted.

**Figure 1:**
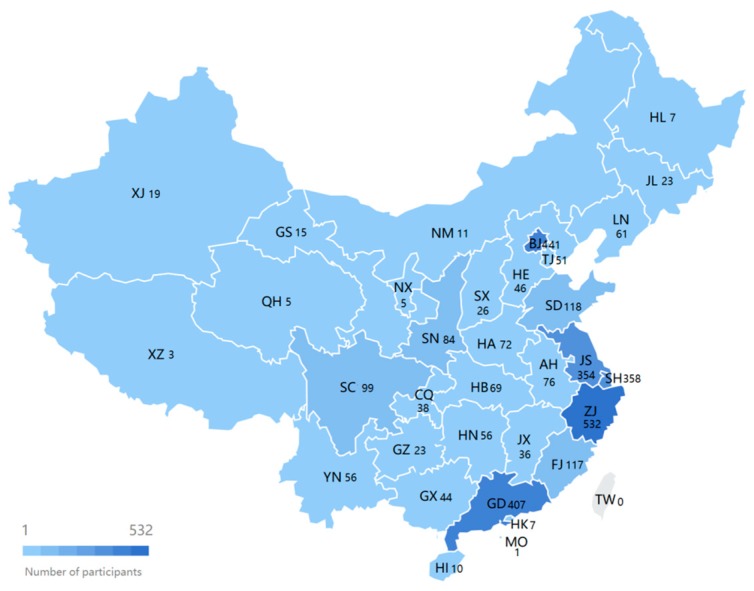
Distribution of participants.

A total of 4604 Chinese users of Bong II were recruited. The data of participants’ daily activities were collected from July 2015 to October 2015. The inclusion criteria were as follows: (1) participants aged 18 years or older; (2) wearing time ≥18 h/day; (3) wear duration ≥4 consecutive days; and (4) data of Global Positioning System (GPS) and air quality are available. The data of 3270 participants were finally included in the analysis. Figure 1 shows the number of participants in each province. The abbreviated names of provinces are in accordance to the authority file released by the Ministry of Industry and Information Technology of China [42]. The research protocol was reviewed and approved by the Ethics Committee of College of Education at Zhejiang University.

### 2.2. Measurement of Sedentary Behaviour

The sedentary time was measured using Bong II. It is 14 g in weight, 4 cm in length, 2 cm in width and 0.9 cm in thickness; it has an adjustable wristband and will not interfere with the participants’ activities. The product is waterproof, and the participants were encouraged to wear it on the nondominant wrist for 24 h a day. The price of Bong II is approximately 20 dollars.

The monitor has a triaxial accelerometer with a sampling frequency of 100 Hz and an output data rate of 1 to 15 s epoch based on the type of activities. The Bong software 2.0 was used to convert the raw 100 Hz files to 15 s epoch files containing x, y and z vectors (mean acceleration over the epoch, retaining the gravity vector) and vector magnitude values (summed over the epoch, corrected for gravity).

The algorithm applied in this study came from Sedentary Sphere, the efficacy of which has been proven valid and reliable for the classification of posture from a wrist-worn triaxial accelerometer in adults [39,41]. The details of the method were mentioned in previous studies [39,41]. Briefly, Sedentary Sphere follows a principle based on arm elevation to identify postures. If the data plotted on Sedentary Sphere in latitudes of elevation are greater than 15° below the horizontal position, this finding indicates that the wrist is elevated. Meanwhile, if the vector magnitude of the acceleration data suggests low intensity of activities, then a sitting or reclining posture can be identified. Meanwhile, if the data plotted on Sedentary Sphere in latitudes of elevation are less than 15° below the horizontal position during low physical activity level, then a standing position is indicated.

The 15 s epoch files were imported into the custom-built Excel spreadsheet developed by Leicester-Loughborough Diet, Lifestyle and Physical Activity Biomedical Research Unit [43], enabling the calculation of the most likely posture with the data of wrist triaxial accelerometers. The validity of measuring sedentary time in free-living adults by a wrist-worn triaxial accelerometer with Sedentary Sphere has been confirmed in several studies given the consistency of its results with those of activPAL, a small lightweight triaxial accelerometer generally considered to be the ‘gold standard’ in identifying sedentary behaviour [39,40,41,44,45]. The intraclass correlation coefficient (ICC) was reported to be 0.80 (95% confidence interval, 0.68–0.88) [40].

In the preliminary research on the reliability and validity of the activity tracker, 32 undergraduate students (18 males and 14 females) were required to wear the activity tracker on their nondominant wrist and activPAL on their right thigh for seven consecutive days in free-living settings, and the ICC between the sedentary time measured by the monitor and activPAL was 0.78 (95% confidence interval, 0.68–0.86) with a mean bias of +18 min. In a laboratory setting, the participants performed a series of required activities, and the accuracy of the wrist-worn activity tracker in classifying lying, sitting and upright activities was 89%. The sensitivity and specificity were 91.8% and 86.3%, respectively.

### 2.3. Air Quality

Daily data of air quality were collected from the data released by the China National Environmental Monitoring Centre and matched in accordance with GPS and time information recorded by the activity tracker. Data of air quality include the daily AQI used by the Chinese government [23], the 24 h average concentrations of particulate matter with aerodynamic diameters less than 10 and 2.5 µm (PM_10_ and PM_2.5_) and the daily maximum 8 h average concentrations of ozone (O_3_).

The AQI was initially divided into six categories according to the recommendation of the Ministry of Environmental Protection of China: excellent (AQI = 0–50), good (AQI = 51–100), lightly polluted (AQI = 101–150), moderately polluted (AQI = 151–200), heavily polluted (AQI = 201–300) and severely polluted (AQI > 300). However, the three most-polluted categories were combined into one because of the paucity of heavily polluted and severely polluted days. Therefore, in this analysis, the AQI was finally divided into four categories as follows: excellent (AQI = 0–50), good (AQI = 51–100), lightly polluted (AQI = 101–150) and moderately and heavily polluted (AQI > 150). Concentrations of air pollutants were also divided into four categories in accordance with the AQI.

### 2.4. Covariates

Age was divided into four categories (18–29, 30–39, 40–49 and above 50). Height and weight were used to determine body mass index (BMI) levels.

Given the evidence that air pollution differs between weekends and weekdays [46] and sedentariness may also differ in a similar pattern [47], a weekday/weekend classification was listed in the potential covariates. The region is also a possible covariate. Thus, provinces were divided into the north and south by a most widely accepted boundary in China, which is intensively applied in studies investigating the regional differences [48,49].

Daily data of ambient temperature and weather were obtained from the China Central Meteorological Observatory and matched in accordance with GPS and time information. The temperature was categorised into quartiles. Weathers were divided into sunny, cloudy and overcast and rainy days.

As variations in wearing time of the activity tracker and wake time are likely to cause differences in recorded sedentary time, monitored wake time was added as a covariate in the analyses.

### 2.5. Statistical Analysis

Two-level linear regression analyses were conducted to investigate the association of air quality and sedentary time. The records of each participant in each day were nested within individuals. Between days, the association between air quality and sedentary behaviour was anticipated to exhibit temporal autocorrelation. Thus, the repeated covariance type was set as first-order autoregressive structure. We used restricted maximum likelihood estimation of variance components.

Firstly, a null model was conducted to determine the necessity of multilevel analysis. Secondly, we investigated the unadjusted and adjusted associations between sedentary time and individual factors, including age, gender, BMI and region, and other potential covariates, such as weather, temperature and weekday/weekend classification. Finally, both unadjusted and adjusted associations between sedentary time and air quality were investigated. The full model included all variables mentioned above. All statistical analyses were conducted using IBM SPSS Statistics v20.0 with a significance level of *p* = 0.05.

## 3. Results

### 3.1. Sedentary Time and Potential Covariates

The descriptive characteristics of 3270 included participants are shown in Table 1. A total of 2021 males and 1249 females were included in this study. The mean age was 30.54 years with a range from 18 years to 86 years. The majority of participants had a normal weight. The mean monitored duration was 11.43 days, longer than the required monitored time in most previous studies [50,51,52,53]. A person-day means a monitored day of a participant, and a total of 37,361 person-days were recorded, which is the sum of the numbers of monitored days of all participants. The mean sedentary time in males was 585.56 min/day, slightly higher than 562.65 min/day in females.

The mean sedentary time per day was 585.56 min (standard deviation (SD): 149.22) in men and 562.65 min (SD: 141.94) in women based on the data collected by the activity tracker. Chinese adults spent nearly 10 h per day on sedentary behaviour. Table 2 shows the associations between sedentary time and potential covariates. The results show that significant association exists between gender and sedentary time (min/day). In the adjusted models, women spent approximately 25 min lesser sedentary time than men (*p* = 0.000). Weather is also significantly associated with sedentary time. On rainy days, sedentary time was 6.73 min longer than sunny days and 5.84 min longer than cloudy and overcast days (*p* = 0.003 and 0.002, respectively). Moreover, sedentary time was 47.43 min longer in weekdays compared with weekends (*p* = 0.000).

### 3.2. Air Quality and Sedentary Time

The ambient air quality during monitored days is presented in Table 3. PM_2.5_, PM_10_ and O_3_ are the major air pollutants in China. The mean concentrations of PM_2.5_ at 24 h, PM_10_ at 24 h, and O_3_ at 8–24 h are 38.69, 64.02 and 124.43 µg/m^3^, which are higher than the national primary limits (10.54%, 28.04% and 24.43%, respectively) [23]. In the majority of the monitored days, the air quality was excellent (31.3%) and good (51.1%). However, several of the monitored days were still lightly polluted (16.5%) and moderately and heavily polluted (1.2%).

Table 4 reports both unadjusted and adjusted associations between sedentary time and air quality. The results indicate a significant association between sedentary time and air quality. In days with excellent, good and lightly polluted air quality, people spent approximately 20 min lesser sedentary time than in moderately and heavily polluted days (Table 4). The sedentary time in days when the concentration of PM_2.5_ is above 115 µg/m^3^ was approximately 45 min longer than in other days with lower concentration of PM_2.5._

## 4. Discussion

The objective of this study was to investigate the association between air quality and sedentary time in Chinese adults, providing evidence for future environmental risk assessment and health behaviour intervention. A wrist-worn activity tracker with an algorithm modified from Sedentary Sphere was utilised to monitor sedentary time. Multilevel analyses were conducted with adjustment for individual factors, temperature, weather and weekday/weekend classification.

The results indicated that air quality was independently associated with sedentary time in Chinese adults. This finding corresponds to several previous studies to some extent. For example, Hu et al. reported that people’s participation in outdoor exercise decreases as air pollution severity increases [31], and Salmon et al. determined that air pollution was significantly associated with an increase in sedentary time [34]. This result also supported the assumption of the socio-ecological model that air quality is an important physical environmental correlate of sedentary behaviour [11]. There are several possible explanations for this finding. Directly, air pollutant inhalation would impair vascular and lung function and decrease exercise performance [54,55], leading to less active time. Indirectly, smog appearance [30], media alerts [27] and mood [13] could play an intermediary role in this relationship. When the ambient air quality is poor, people may cancel their plans of outdoor activity and instead stay indoors sedentarily because of concerns about the potential negative effects of air pollution on health [56]. Further studies are needed to explore more plausible mechanisms.

The finding suggests that better AQI levels were significantly associated with approximately 20 min reduction in sedentary time, and lower levels of PM_2.5_ pollution were associated with approximately 45 min reduction in sedentary time. We compared these magnitudes of effects with those of several other environmental factors of sedentary time in previous studies. Compared with long day length and good weather conditions, short day length and poor weather conditions, including high precipitation and low temperatures, were associated with 15 min longer sedentary time [57]. Notably, women living in medium- and high-walkable neighbourhoods reported significantly 14 and 17 min less TV viewing time per day compared with those residing in low-walkable neighbourhoods [19]. This comparison indicated that the magnitude of the effect of air quality may be equal to or larger than some other factors at the environmental level.

According to the results of this study, people tend to prolong their sedentary time in moderately and heavily polluted days. Therefore, in these days, it is particularly necessary to remind people not to spend too much time on sedentary behaviour and perform more other indoor activities. Given that air quality is an important factor of sedentary time, the government and the public should make efforts to reduce air pollution not only for its direct impairment on health [25,26] but also for its association with unhealthy behaviours. However, considering that the exact amount of sedentary time a person would have to reduce in order to achieve meaningful health benefits is still unclear due to the preference to categorisation of sedentary time in previous studies [58], further studies are needed to investigate whether the magnitudes of effects of improved air quality are large enough to help people gain a significantly decreased mortality.

Admittedly, this study presents limitations. Firstly, the map (Figure 1) shows that the sample is not balanced amongst provinces. The majority of participants were in the east and the middle. Thus, the sample might not be entirely representative for the entire country. However, participants were from 33 out of 34 provinces, autonomous regions and municipalities of China, and the majority of Chinese population reside in the east and the middle [59]. Thus, the participants in this study could still reflect the current situation of Chinese adults to some extent. Secondly, although the sample size was relatively large, the monitored days were unevenly distributed in each air quality level. Moreover, buyers of the wrist-worn activity tracker could share several common characteristics, such as socio-economic status and exercise habits, such that the participants are not entirely representative of all Chinese citizens. In addition, this study is unable to distinguish contexts of sedentary behaviour, such as occupational, traffic-related and leisure sedentariness, which limits further analyses. Finally, one limitation of Sedentary Sphere is that its classification accuracy for sitting postures was reported to be approximately 60%, with the majority of misclassifications occurring during the sitting postures that did not involve any accompanying hand movement [39,41]. However, although the accuracy rate of Sedentary Sphere is lower than that of activPAL and ActiGraph, the effects of this weakness can be attenuated if the sample size is sufficient. Regardless, the relationship between air quality and sedentary behaviour requires further investigation with the assistance of novel data processing technologies.

The strengths of this study lay in its novel posture determination algorithm, relatively large sample size, well-matched air quality data and multilevel analyses. Firstly, instead of determining sedentary time merely based on energy expenditure, Sedentary Sphere was applied and assisted in estimating sedentary time. In addition, the affordable cost of this commercial wrist-worn activity tracker with acceptable validity allowed a relatively large sample size in our study. A total of 3270 participants from 33 of 34 provinces in China were included in the analysis. However, only few previous studies applied this novel technology. The application of Sedentary Sphere in the current study showed its potential to expand more possibilities for future studies. Moreover, in previous studies investigating the relationship between air quality and physical activity, self-reported physical activity data were commonly matched to monthly average air quality parameters. By contrast, in the current study, daily data of sedentary time and parameters of 24 h air quality where sedentary behaviours occurred were accurately matched, leading to better precision. Besides, unlike previous studies [27,28,29,31], we used multilevel modelling including a within-subject component, which increases the power of this study and helps make more causal interpretations.

## 5. Conclusions

Air quality appears to be an important factor associated with sedentary time of Chinese adults. People spent significantly longer sedentary time in moderately and heavily polluted days than in other days with better air quality.

## Figures and Tables

**Table 1:** Descriptive information of the study participants.

	Total	Male	Female	*t*/*χ*^2^	*p*
Number of participants (%)	3270 (100)	2021 (61.8)	1249 (38.2)	-	-
Person-day	37,361	23,225	14,136	-	-
Wearing time/day, hour; mean (SD)	22.69 (0.98)	22.67 (1.00)	22.71 (0.94)	−4.69 ***	0.000
Monitored duration, day; mean (SD)	11.43 (4.15)	11.49 (4.11)	11.32 (4.20)	1.17	0.244
Age group (%)					
18–29	1763 (53.9)	1089 (53.9)	674 (54.0)	4.86	0.182
30–39	1171 (35.8)	723 (35.8)	448 (35.9)		
40–49	247 (7.6)	145 (7.2)	102 (8.2)		
Above 50	89 (2.7)	64 (3.2)	25 (2.0)		
BMI level (%)					
Underweight	757 (23.1)	376 (18.6)	381 (30.5)	104.04 ***	0.000
Normal	2175 (66.5)	1386 (68.6)	789 (63.2)		
Overweight	327 (10.0)	258 (12.8)	69 (5.5)		
Obese	11 (0.3)	1 (0.0)	10 (0.8)		
Sedentary time/day, minute; mean (SD)	576.89 (146.93)	585.56 (149.22)	562.65 (141.94)	14.84 ***	0.000

*** *p* < 0.001.

**Table 2:** Associations between sedentary time (minutes) and potential covariates.

		Unadjusted			Adjusted ^1^		
		Estimate (SE)	95% CI	*p*	Estimate (SE)	95% CI	*p*
Age	18–29	12.51 (9.88)	−6.86, 31.89	0.205	9.76 (10.81)	−11.45, 30.96	0.367
	30–39	4.04 (10.00)	−15.57, 23.64	0.686	0.54 (10.91)	−20.87, 21.94	0.961
	40–49	−6.73 (11.27)	−28.83, 15.37	0.550	−7.40 (12.32)	−31.58, 16.77	0.548
	≥50 (ref)	-	-	-	-	-	-
Gender	Female	−21.90 (3.29) ***	−28.35, −15.45	0.000	−24.69 (3.71) ***	−31.96, −17.43	0.000
	Male (ref)	-	-	-	-	-	-
BMI	Underweight	−13.36 (28.67)	−69.57, 42.85	0.641	−53.87 (33.49)	−119.53, 11.79	0.108
	Normal	−12.95 (28.54)	−68.92, 43.00	0.650	−60.57 (33.36)	−125.99, 4.85	0.070
	Overweight	−11.19 (28.93)	−67.91, 45.53	0.699	−60.02 (33.78)	−126.27, 6.22	0.076
	Obese (ref)	-	-	-	-	-	-
Region	North	−5.77 (3.50)	−12.63, 1.10	1.00	−6.51 (4.12)	−14.60, 1.58	0.115
	South (ref)	-	-	-	-	-	-
Weather	Sunny	−8.04 (2.30) ***	−12.55, −3.52	0.000	−6.73 (2.28) **	−11.24, −2.23	0.003
	Cloudy and overcast	−3.67 (1.89)	−7.39, 0.05	0.053	−5.84 (1.88) **	−9.53, −2.16	0.002
	Rain (ref)	-	-	-	-	-	-
Weekday/weekend	Weekdays	48.57 (1.44) ***	45.74, 51.39	0.000	47.43 (1.69) ***	44.12, 50.74	0.000
	Weekends (ref)	-	-	-	-	-	-
Temperature	<20	2.95 (3.18)	−3.27, 9.18	0.353	−0.57 (3.49)	−7.42, 6.28	0.870
	20–22.1	10.51 (2.76) ***	5.09, 15.92	0.000	5.54 (3.06)	−0.47, 11.55	0.071
	22.1–25	6.12 (2.51) *	1.20, 11.05	0.015	4.13 (2.76)	−1.29, 9.54	0.135
	≥25 (ref)	-	-	-	-	-	-

^1^ The adjusted model included all seven variables. * *p* < 0.05, ** *p* < 0.01, *** *p* < 0.001

**Table 3:** Descriptive statistics of air quality ^1^.

**AQI Categories, Days (%)**	
Excellent (AQI = 0–50)	11,680 (31.3)
Good (AQI = 50–100)	19,088 (51.1)
Lightly polluted (AQI = 100–150)	6155 (16.5)
Moderately and heavily polluted (AQI > 150)	438 (1.2)
**PM_2.5_ Levels, Days (%)**	
0–35 µg/m^3^	18,594 (49.8)
35–75 µg/m^3^	16,485 (44.1)
75–115 µg/m^3^	2112 (5.7)
>115 µg/m^3^	170 (0.5)
**PM_10_ Levels, Days (%)**	
0–50 µg/m^3^	13,087 (35.0)
50–150 µg/m^3^	23,630 (63.2)
150–250 µg/m^3^	626 (1.7)
>250 µg/m^3^	18 (0.0)
**O_3_ Levels, Days (%)**	
0–160 µg/m^3^	23,960 (64.1)
160–200 µg/m^3^	7716 (20.7)
200–300 µg/m^3^	5423 (14.5)
>300 µg/m^3^	262 (0.7)

^1^ The classification of AQI and concentrations of air pollutants are according to the Ambient Air Quality Standards in China.

**Table 4:** Associations between air quality and sedentary time (minutes).

		Unadjusted			Adjusted ^1^		
		Estimate (SE)	95% CI	*p*	Estimate (SE)	95% CI	*p*
AQI	Excellent	−12.21 (6.56)	−25.08, 0.65	0.063	−19.20 (8.63) *	−34.91, −3.49	0.017
	Good	−17.44 (6.51) **	−30.20, −4.69	0.007	−22.92 (7.94) **	−38.48, −7.35	0.004
	Lightly polluted	−9.10 (6.48)	−21.79, 3.60	0.160	−21.28 (7.91) **	−36.78, −5.78	0.007
	Moderately and heavily polluted (ref)	-	-	-	-	-	-
PM_2.5_	0–35 µg/m^3^	−36.80 (10.64) ***	−57.65, −15.94	0.001	−45.35 (12.21) ***	−69.29, −21.41	0.000
	35–75 µg/m^3^	−36.77 (10.63) ***	−57.61, −15.93	0.001	−45.74 (12.19) ***	−69.64, −21.84	0.000
	75–115 µg/m^3^	−33.13 (10.80) **	−54.29, −11.96	0.002	−45.54 (12.35) ***	−69.74, −21.33	0.000
	>115 µg/m^3^ (ref)	-	-	-	-	-	-
PM_10_	0–50 µg/m^3^	−32.81 (32.87)	−97.24, 31.62	0.318	−32.59 (32.60)	−96.49, 31.31	0.318
	50–150 µg/m^3^	−36.31 (32.85)	−100.68, 28.07	0.269	−35.65 (32.57)	−99.48, 28.18	0.274
	150–250 µg/m^3^	−26.47 (32.92)	−90.99, 38.06	0.421	−31.07 (32.66)	−95.08, 32.94	0.341
	>250 µg/m^3^ (ref)	-	-	-	-	-	-
O_3_	0–160 µg/m^3^	−3.74 (8.25)	−19.83, 12.34	0.648	−6.17 (10.57)	−26.89, 14.55	0.560
	160–200 µg/m^3^	−2.68 (8.30)	−18.94, 13.58	0.747	−6.14 (10.63)	−26.98, 14.69	0.563
	200–300 µg/m^3^	3.93 (8.16)	−12.05, 19.92	0.629	−5.53 (10.50)	−26.11, 15.04	0.598
	>300 µg/m^3^ (ref)	-	-	-	-	-	-

^1^ Adjusted for gender, weather, temperature, weekday/weekend and monitored wake time per day. * *p* < 0.05, ** *p* < 0.01, *** *p* < 0.001.
